# Small for gestational age infants achieve physiologic milestones related to discharge at a later postmenstrual age

**DOI:** 10.1038/s41372-024-02059-2

**Published:** 2024-07-20

**Authors:** Adriana C. Hoffman, Sarah D. Ronis, Rita M. Ryan

**Affiliations:** Rainbow Babies and Children’s Hospital, Cleveland, OH USA

**Keywords:** Ageing, Risk factors

In a prior study to examine the impact of requiring a minimum weight of 1800 grams (g) for discharge from our neonatal intensive care unit, we found that in a cohort of babies with birthweight 1300–1800 g, small for gestational age (SGA) infants had a significantly later (~1 week (w) higher) postmenstrual age (PMA) at the time of physiologic maturity/discharge readiness [1]. The American Academy of Pediatrics outlines discharge readiness in preterm infants by meeting several competencies [2]. We specifically defined these competencies as five discrete measurable milestones, based on our discharge practices. In the previous study, we had also relied on obstetrical best dating estimate for gestational age (GA), but not all mothers had a first-trimester ultrasound. Since this was an incidental secondary finding previously, we performed a new study to test the hypothesis that accurately dated premature SGA infants reach physiologic maturation later than appropriate for GA (AGA) infants.

We performed a retrospective chart review of all SGA infants, GA confirmed by first-trimester ultrasound, born from 2021–2022 < 37w with birthweight <10th percentile and compared to GA-matched AGA control infants (Fig. S1). Each infant’s chart was reviewed to assess the day of life and PMA when physiologic maturity was reached based on the following milestones: (1) stable cardiorespiratory function defined as no apneas or bradycardias for 5 days without methylxanthines and (2) no unprompted desaturations for 24 h without supplemental oxygenation; (3) adequate maintenance of body temperature fully clothed with normal ambient temperature (20–25 °C), for 48 h in an open crib; (4) competent feeding without cardiorespiratory compromise, for which we measured oral feeding of ≥120 ml/kg/day without gavage feedings OR eight breastfeeding sessions without complications noted by lactation consultant without gavage feedings; and (5) sustained pattern of weight gain, which we measured as weight gain for a minimum of 2 days on only oral feeding. We charted the date and PMA for achieving each and all five milestones, which was considered as having achieved physiologic maturity, and discharge readiness. All charts were reviewed by one person (ACH) for consistency. Demographic information collected is included in Fig. 1A. There was an even number of congenital anomalies in both the SGA and AGA groups. However, infants who were deceased prior to discharge were excluded. A sample size calculation based on the one-week difference we found previously, indicated we needed 26 babies in each group. Since we were including all birthweights and only those with accurate dating which might change these sample size assumptions, we increased our sample collection and analyzed all SGA preterm infants born between 2021-2022, and then matched for GA and closest birthdate.

**Fig. 1:**
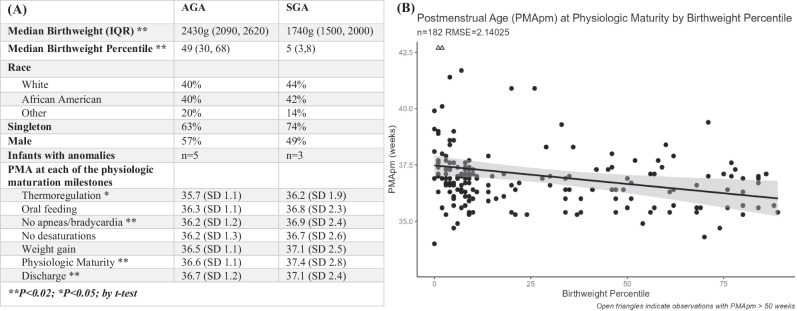
As discrete variables, thermoregulation, resolution of apneas and bradycardias, and attainment of physiologic maturty as having reached all 5 milestones was signifcantly delayed in SGA infants. SGA infants were discharged from the hospital at a later PMA. As a continous variable, lower birthweight percentile was associated with acieving physiologic maturity at a later PMA. **A** Demographic and characteristic information on infants studied and PMA of milestone achievements by GA classification. Congenital anomalies included VACTERL, duodenal web, TAPVR, gastroschisis, neonatal encephalopathy, congenital diaphragmatic hernia and imperforate anus. **B** Postmenstrual age at physiologic maturity plotted by birthweight percentile via linear regression with confidence interval shaded along regression. For every decrease in 1 in BW percentile, PMA at physiologic maturity increased by 0.02 days. BW percentile and PMA at physiologic maturity were significantly associated (linear regression (*p* = 0.0038)).

We analyzed 182 charts. The median GA of all infants included was 34.9w (IQR 33.9–36.3). Median BW percentile for SGA was 5 (IQR 3–8), compared to AGA 49 (IQR 30–68). Mean PMA at physiologic maturity for AGA infants was 36.6w compared to 37.4w for SGA infants, making a difference of 0.8w or 5.6 days. Median PMA at physiologic maturity for SGA was 37w (IQR 36.4–37.6), compared to AGA 36.6w (IQR 35.7–37.1) (Fig. S2). When looking at BW as a continuous variable, for every decrease in 1 BW percentile, PMA at physiologic maturity increased by 0.02 days (*P* = 0.004, CI 95% −0.028 to 0.005) (Fig. 1B). Looking at each individual milestone, mean PMA was consistently increased for SGA compared to AGA, with a significant increase in thermal regulation and no apnea/bradycardia for 5 days (Fig. 1A) by *t*-test.

In conclusion, SGA infants reached physiologic maturity and were discharged significantly later than AGA infants. The later discharge could be explained by lower weight but the reason for the delay in achieving physiologic maturity is unclear. We did not examine the identified reason (if any) for fetal growth restriction. There was increased variation in the SGA group as compared to the AGA, which could reflect a mixture of fetal growth restriction, maternal factors and constitutionally small infants. Customized fetal growth charts have shown limited ability to predict adverse outcomes [3], but may be a direction for future studies to better characterize the relationship between SGA status and delayed physiologic maturity. Although we considered our definitions of each milestone to be objective, there may be some bias for babies of lower birthweight as to how these events are documented, how aggressively babies are fed, etc., particularly in a unit with a minimum-weight discharge policy. We believe this is the first study to report this association of delayed maturation with SGA status and decreased BW percentile. This information may be helpful for discharge planning for SGA infants, for hospital cost analyses or further research studies.

## Supplementary information

Figure S1

Figure S2

Supplementary Figure Legends
