# Supplementary figures and images for: Small for gestational age infants achieve physiologic milestones related to discharge at a later postmenstrual age
Source: J Perinatol. 2024 Jul 20;45(3):402–3. doi: 10.1038/s41372-024-02059-2 (PMC11888982; doi:10.1038/s41372-024-02059-2)

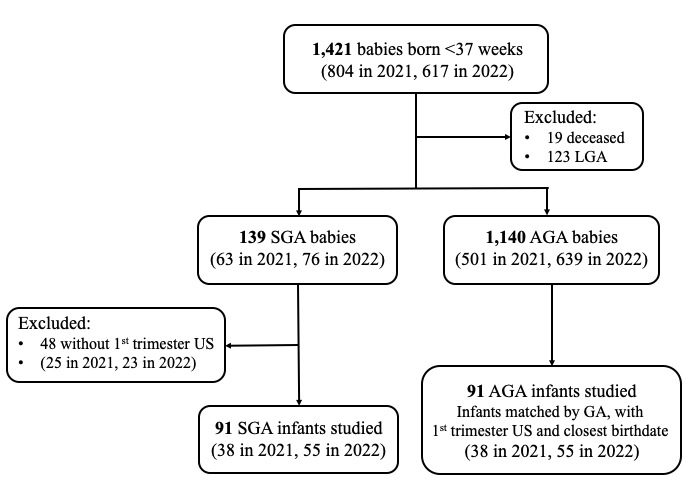

Supplement: Supplementary file 1 — Figure S1 [file 41372_2024_2059_MOESM1_ESM.docx]

**
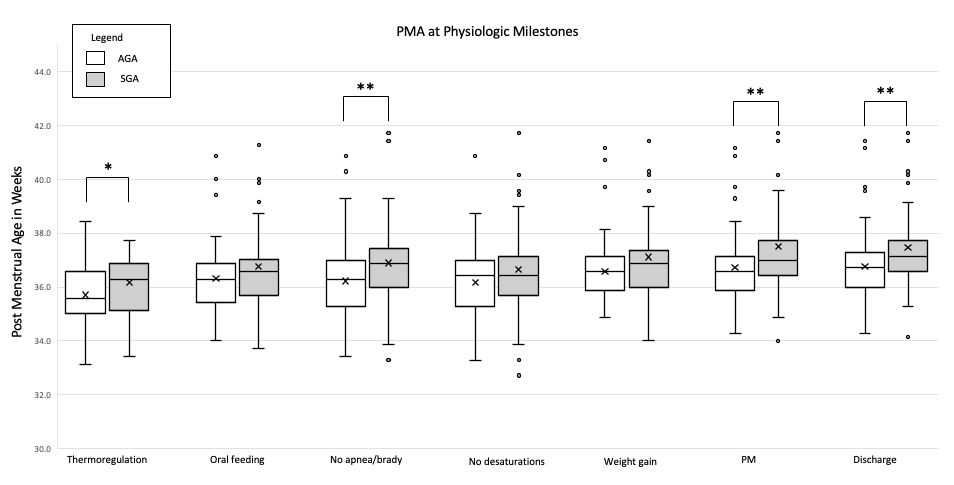
**

Supplement: Supplementary file 2 — Figure S2 [file 41372_2024_2059_MOESM2_ESM.docx]
