# Supplementary material for: Small for gestational age infants achieve physiologic milestones related to discharge at a later postmenstrual age
Source: J Perinatol. 2024 Jul 20;45(3):402–3. doi: 10.1038/s41372-024-02059-2 (PMC11888982; doi:10.1038/s41372-024-02059-2)
Supplement: Supplementary file 3 — Supplementary Figure Legends [file 41372_2024_2059_MOESM3_ESM.docx]

**Figure S1.** Consort diagram showing participant eligibility and final infants included in study, differentiated by calendar year

**Figure S2.** Box and whisker plot of mean postmenstrual age (by weeks) for each of the milestones measured for AGA vs SGA infants; Thermoregulation (maintaining body temperature with normal ambient temperature (20–25°C) for 48 hours in an open crib); Oral feeding (taking >120ml/kg/day of formula/breastmilk or breastfeeding without issues noted by lactation, without gavage feedings); No apnea/brady (no apneas or bradycardias for five days without methylxanthines); No desaturations (No desaturation <80% that were unprompted for at least 24 hours without supplemental oxygen); Weight gain (two consecutive days of weight gain, exclusively on oral feeding); Physiologic maturity defined as attainment of all five defined milestones, and PMA at actual discharge date. X marking the mean for each millstone. (**P<0.02; *P<0.05; by t test).
